# Chunghyul-dan, a multi-botanical ethanol extract, improves collateral perfusion and neurovascular stability in permanent focal cerebral ischemia

**DOI:** 10.3389/fphar.2026.1736412

**Published:** 2026-04-20

**Authors:** Han-Gyul Lee, Tae Woo Kwon, Minho Jung, Won Myoung Lee, Chang-Dae Lee, Seungwon Kwon, Sanghyun Lee, Ik-Hyun Cho, Sang-Kwan Moon

**Affiliations:** 1 Department of Cardiology and Neurology, College of Korean Medicine, Kyung Hee University, Seoul, Republic of Korea; 2 Department of Convergence Medical Science, College of Korean Medicine, Kyung Hee University, Seoul, Republic of Korea; 3 Department of Science in Korean Medicine, Graduate School, Kyung Hee University, Seoul, Republic of Korea; 4 Department of Plant Science and Technology, Chung-Ang University, Anseong, Republic of Korea; 5 Natural Product Institute of Science and Technology, Chung-Ang University, Anseong, Republic of Korea; 6 Institute of Convergence Korean Medicine, College of Korean Medicine, Kyung Hee University, Seoul, Republic of Korea

**Keywords:** angiogenesis, Chunghyul-dan, collateral circulation, ischemic stroke, neuroinflammation

## Abstract

**Background:**

Collateral perfusion and neurovascular stability critically influence outcomes after ischemic stroke; however, no pharmacological agent is currently approved to enhance these processes.

**Objectives:**

Chunghyul-dan (CHD), a standardized multi-botanical ethanol extract with reported vascular and anti-inflammatory properties, was evaluated for its neurovascular protective effects in a permanent middle cerebral artery occlusion (pMCAO) mouse model.

**The Process and Methods:**

Male ICR mice subjected to pMCAO received oral Chunghyul-dan (30–120 mg/kg). Cortical infarct volume, anterior cerebral artery perfusion, angiogenesis-related signaling, neuroinflammation, and endothelial barrier integrity were assessed using histological analysis, laser Doppler flowmetry, immunohistochemistry, and complementary *in vitro* studies in BV2 microglia and bEND.3 endothelial cells.

**Results:**

CHD significantly reduced cortical infarct volume, with maximal protection observed at 60 mg/kg. Laser Doppler analysis demonstrated enhanced ACA perfusion at 30 mg/kg during early ischemia. CHD upregulated VEGF expression in ischemic cortex and endothelial cells, supporting vascular remodeling. Neuroinflammation was attenuated, as CHD reduced Iba-1–positive microglial activation and suppressed iNOS, IL-6, and TNF-α expression. Endothelial barrier integrity was preserved through reduced PECAM-1 expression, restoration of claudin-5 and occludin, and selective inhibition of STAT3 phosphorylation, whereas TLR4/MAPK signaling remained unchanged.

**Conclusion:**

CHD confers multi-level neurovascular protection by promoting collateral perfusion, facilitating VEGF-associated vascular adaptation, suppressing neuroinflammation, and stabilizing endothelial junctional architecture. These findings support further translational evaluation of CHD as a pharmacological modulator of collateral circulation in ischemic stroke.

## Graphical Abstract

**Figure:**
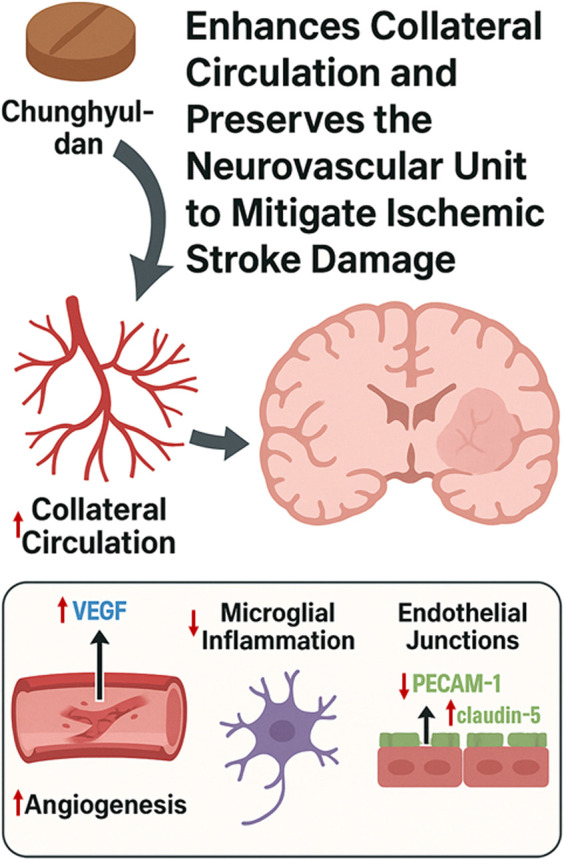
Proposed mechanism of neurovascular protection by CHD. Schematic illustration summarizing the dual actions of CHD in ischemic stroke: (i) enhancement of collateral perfusion through VEGF-mediated angiogenesis and sustained ACA flow, and (ii) suppression of microglial activation, inhibition of pro-inflammatory mediators, and stabilization of endothelial barrier function. CHD reduced PECAM-1 expression and preserved tight junction proteins (claudin-5) via selective inhibition of the STAT3 pathway, thereby maintaining blood–brain barrier integrity.

## 1 Introduction

In 2021, stroke ranked as the third leading cause of death worldwide (7.3 million deaths; 10.7% of all deaths) and the fourth leading cause of disability-adjusted life years (DALYs) (160.5 million DALYs; 5.6% of all DALYs) (Collaborators, 2024). That year, there were an estimated 11.9 million new cases and 93.8 million people living with the consequences of stroke. Resulting from the interruption of cerebral blood flow and subsequent neuronal death, its global burden continues to rise with population aging, and the long-term care of survivors imposes a substantial socioeconomic burden on both individuals and society.

Among stroke subtypes, ischemic stroke accounts for the majority of cases and presents with diverse and complex clinical manifestations. Although reperfusion therapies—such as tissue plasminogen activator (t-PA) administration and mechanical thrombectomy—can achieve favorable outcomes when applied during the hyperacute phase under specific conditions, their clinical utility is constrained by a narrow therapeutic window and strict eligibility criteria (Groan et al., 2021; Fan et al., 2025; Sarraj et al., 2025). As a result, only a small proportion of patients benefit from these interventions, and many survivors experience persistent neurological deficits (Xuan Zheng et al., 2021). These limitations underscore the urgent need for novel therapeutic strategies that extend beyond conventional reperfusion approaches.

Collateral circulation**,** a network of pre-existing arterial anastomoses, plays a critical role in maintaining cerebral perfusion after partial occlusion of major arteries in ischemic stroke (Maguida and Shuaib, 2023). Robust collateral flow is strongly associated with smaller infarct volumes, better functional recovery, and lower mortality. By sustaining residual blood supply to ischemic regions, collateral vessels extend the therapeutic window for intervention and mitigate irreversible tissue injury. Despite advances in understanding the structural and functional dynamics of this vascular bypass system, no pharmacological agent has yet been approved to specifically enhance collateral circulation in stroke patients (Maguida and Shuaib, 2023; Liu et al., 2025; Marilena et al., 2025). Pharmacological modulation of collateral flow therefore represents a promising and complementary approach to invasive intracranial procedures (Vasquez et al., 2021).

Natural product–based therapeutics have attracted increasing attention for their favorable safety profiles and multi-target pharmacological actions in promoting resilience and treating diverse diseases (Li, 2016; Zhu et al., 2022). Chunghyul-dan (CHD), a modified formulation of Hwangryunhaedok-tang (Huanglianjiedu-tang in Chinese, Orengedokuto in Japanese), is a combinatorial botanical drug composed of Scutellariae Radix (*Scutellaria baicalensis* Georgi), Coptidis Rhizoma (*Coptis japonica* Makino), Phellodendri Cortex (*Phellodendron amurense* Rupr.), Gardeniae Fructus (*Gardenia jasminoides* Ellis), and Rhei Rhizoma (*Rheum palmatum* L.) (Kim et al., 2010; Jung et al., 2018). It contains multiple specialized metabolites, including flavonoids (e.g., baicalin, wogonin), alkaloids (e.g., berberine), iridoid glycosides (e.g., geniposide), and dianthrone glycoside (e.g., sennoside A) (Kim et al., 2010). Historically, Hwangryunhaedok-tang has been prescribed to alleviate heat, inflammation, and circulatory disturbances, particularly in disorders involving hypertension, atherosclerosis, and cerebral hyperperfusion (Xiong et al., 2013; Qi et al., 2019; Lee and Kwon, 2020; Jin et al., 2024). Clinical and experimental studies have demonstrated its antihyperlipidemic, anti-inflammatory, vasorelaxant, and cerebrovascular flow–enhancing effects (Xiong et al., 2013; Qi et al., 2019; Lee and Kwon, 2020; Jin et al., 2024). In traditional medicine, Daio-Orengedokuto (Daehwang-Hwangryunhaedok-tang)—a derivative formula supplemented with *Rhei Rhizoma*—has long been used for patients with atherosclerosis accompanied by constipation, a condition associated with increased stroke risk (Kim et al., 2002). Based on these traditional and pharmacological backgrounds, CHD, a modern standardized form of Daio-Orengedokuto, has been developed and investigated for its potential neurovascular protective effects and therapeutic applicability in cerebrovascular diseases.

Previous studies have reported diverse pharmacological effects of CHD. It has been shown to inhibit stroke recurrence by reducing microangiopathy progression in patients with small vessel disease (Jung et al., 2018); protect dopaminergic neurons by suppressing reactive oxygen species (ROS) generation and preventing mitochondrial dysfunction in an *in vitro* Parkinson’s disease model (Kim et al., 2010); and reduce histopathological damage while improving motor and cognitive functions in a mouse traumatic brain injury model, without affecting brain edema or blood–brain barrier (BBB) integrity (Choi et al., 2017). In addition, CHD exhibits antioxidative, anti-inflammatory, antihypertensive, and antilipidemic activities, suggesting that it may preserve endothelial function, promote vasodilation, and facilitate arteriogenesis—mechanisms that could enhance collateral circulation and cerebral blood flow (Fan et al., 2022).

Based on these properties, we hypothesized that CHD enhances collateral circulation, thereby improving cerebral perfusion and mitigating ischemic injury. The present study aimed to evaluate the neuroprotective efficacy and elucidate the underlying mechanisms of CHD in a permanent middle cerebral artery occlusion (pMCAO) mouse model. To our knowledge, this is the first study to demonstrate that CHD reduces cortical infarct volume in ischemic stroke *via* enhancement of cerebral blood flow.

## 2 Materials and methods

### 2.1 Reagents and apparatus

Berberine, baicalin, geniposide, wogonin, and sennoside A were supplied by the Natural Product Institute of Science and Technology, Anseong, Republic of Korea. Chromatographic analysis was performed on a Waters HPLC system equipped with a 1525 Binary HPLC pump and a 2489 UV/Vis detector (Waters, Milford, MA, United States). HPLC-grade acetonitrile (ACN), trifluoroacetic acid, and water were obtained from Scharlau (Barcelona, Spain) and Honeywell (Burdick & Jackson, Muskegon, MI, United States).

### 2.2 Preparation of CHD

CHD is a laboratory-prepared 80% ethanol extract composed of five botanical drugs mixed in a fixed weight ratio (1:1:1:1:1): Scutellariae Radix (*S. baicalensis* Georgi), Coptidis Rhizoma (*C. japonica* Makino), Phellodendri Cortex (*P. amurense* Rupr.), Gardeniae Fructus (*G. jasminoides* Ellis), and Rhei Rhizoma (*R. palmatum* L.), as previously described (Jung et al., 2018; Lee et al., 2023). Each dried botanical drug was purchased from Kyung Hee Herb Pharm (Wonju-si, Gangwon-do, Republic of Korea), and botanical authentication was performed by Prof. Sang Kwan Moon (Kyung Hee University Korean Medicine Hospital). Voucher specimens were deposited in the Herbarium of the same institution. The crude botanical drugs were mixed according to the above ratio and extracted with 80% ethanol under reflux at 100 °C for 2 h. The ratio refers to the weight of crude botanical drugs prior to extraction. The extract was filtered, concentrated under reduced pressure using a rotary evaporator, and lyophilized to obtain a powdered extract, yielding 26.5% (w/w). All extract preparations were manufactured under identical laboratory conditions to ensure reproducibility. All botanical materials were commercially sourced and complied with national regulations. The species used are not listed under CITES and are not subject to Nagoya Protocol restrictions.

### 2.3 Quality control of CHD extract

*Sample and Standard Preparation:* For analysis, sample aliquots were dissolved in 80% methanol (MeOH) to a concentration of 20 mg/mL and subsequently clarified through a 0.45 μm PVDF syringe filter. A series of working standard solutions were prepared by the systematic dilution of a stock solution (also in 80% MeOH) to establish calibration curves. *Chromatographic Analysis:* A YMC-Pack Pro C18 column (4.6 × 250 mm, 5 μm) was used for the analysis at a column temperature of 35 °C. Detection was performed at a wavelength of 270 nm with an injection volume of 10 μL. The mobile phase, comprising 0.1% trifluoroacetic acid in water (A) and ACN (B), was pumped at 1.0 mL/min. The elution gradient was programmed as follows: an initial isocratic step at 7% B for 10 min, followed by a linear increase to 60% B over the next 35 min. The concentration of B was then rapidly increased to 90% within 1 min and held for 4 min. Finally, the column was re-equilibrated at the initial 7% B for 10 min prior to the subsequent run.

### 2.4 Animals and housing conditions

Seven-to eight-week-old male ICR mice (29–30 g; Samtako BIO KOREA Co., Ltd., Osan, Republic of Korea) were housed in standard polycarbonate cages (five per cage) under controlled conditions (12-h light/dark cycle, lights on at 07:00; temperature 23 °C ± 3 °C; relative humidity 55% ± 5%). Food and water were provided *ad libitum*. Animals were acclimated for at least 1 week before experimentation.

### 2.5 Ethics statement

All experimental procedures involving animals were performed in accordance with the ethical guidelines for the care and use of laboratory animals and received prior approval from the Institutional Animal Care and Use Committee of Kyung Hee University (KHUASP(SE)-23-488). Randomization and blinded data analysis were performed in accordance with NIH guidelines for rigor in preclinical neurological research (Landis et al., 2012). Every effort was made to minimize animal use and suffering during the experiments.

### 2.6 Administration of CHD

CHD and the vehicle control were administered once *via* oral gavage 10 min prior to surgery. CHD was given at doses of 30, 60, and 120 mg/kg, calculated based on the human equivalent dose (HED) and adjusted for mice using standard body surface area conversion methods. The vehicle group received an equivalent volume of sterile distilled water (DW).

### 2.7 Permanent middle cerebral artery occlusion (pMCAO) surgery

The pMCAO model was established using a modified protocol previously described (Majid et al., 2000). Male ICR mice (29–30 g) were anesthetized with 2% isoflurane in a 70% N_2_O and 30% O_2_ mixture, and anesthesia depth was monitored by loss of reflex to toe pinch. After placing the animal in a stereotaxic frame, the scalp was incised between the left eye and ear, and the temporalis muscle was retracted to expose the temporal bone. A 2.0 mm burr hole was carefully drilled over the distal segment of the left middle cerebral artery (MCA) using a high-speed microdrill under a surgical microscope. Following dural removal, the main trunk of the distal MCA was coagulated using a bipolar coagulator and subsequently transected with micro-scissors to ensure permanent occlusion. Mice exhibiting subarachnoid hemorrhage, failure to develop neurological symptoms (e.g., ipsilateral circling), or mortality during surgery were excluded from analysis. Body temperature was maintained at 36.5 °C–37.5 °C using a thermostatically controlled heating pad (Harvard Apparatus, Holliston, MA, United States) throughout the surgical procedure, and no animals were allowed to drop below 36 °C. To ensure reproducibility, all surgeries were performed by the same experienced microsurgeon, and procedures were conducted under blinded and randomized conditions in accordance with NIH recommendations for preclinical neurological studies (Landis et al., 2012).

### 2.8 Cerebral blood flow (rCBF) monitoring by laser doppler flowmetry

**FIGURE 1:**
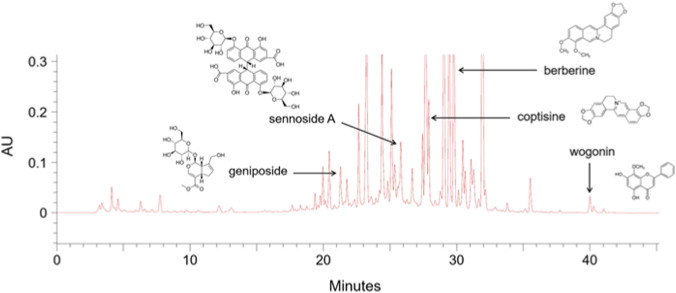
Phytochemical profile of Chunghyul-dan (CHD). Representative HPLC chromatogram obtained using a YMC-Pack Pro C18 column (4.6 × 250 mm, 5 μm; 35 °C) with detection at 270 nm. The mobile phase consisted of 0.1% trifluoroacetic acid in water and acetonitrile at a flow rate of 1.0 mL/min under a gradient elution (7%–60% B, 0–35 min). Major marker compounds were identified by co-elution with authentic standards: geniposide, sennoside A, coptisine, berberine, and wogonin. These peaks confirm the reproducible phytochemical profile of CHD used in this study.

To assess the collateral blood flow dynamics before and after pMCAO, regional rCBF was measured using Laser Doppler flowmetry (moorVMS-LDF2, Moor Instruments Ltd., United Kingdom). A flow probe was affixed to the skull using cyanoacrylate adhesive at a site corresponding to the distal ischemic region (1.5 mm lateral to the midline and 1.0 mm posterior to bregma). Real-time monitoring of anterior cerebral artery (ACA) blood flow was conducted using the moorVMS-PC software (Moor Instruments Ltd., United Kingdom). Baseline rCBF was recorded prior to vessel coagulation. Measurements were continuously collected for 15 min following occlusion of the distal MCA. Changes in blood flow were quantified as percentage values relative to pre-occlusion baseline at 5, 10, and 15 min post-occlusion. These measurements allowed the evaluation of early collateral perfusion and the efficacy of pharmacological intervention (Figure 1).

### 2.9 Tissue preparation for histological analysis

Tissue preparation was performed based on established protocols for histological evaluation after focal cerebral ischemia (Oh et al., 2024; Ha et al., 2025; Oh et al., 2025). Briefly, mice were deeply anesthetized 24 h after pMCAO and transcardially perfused with 0.1 M phosphate-buffered saline (PBS) followed by 4% paraformaldehyde (PFA). Brains were post-fixed in 4% PFA at 4 °C for 24 h and subsequently cryoprotected in 30% sucrose solution for at least 3 days. Coronal brain sections (30 μm thick) including the infarct core were obtained using a cryostat (CM3050S, Leica Biosystems, Germany) and stored in PBS until use.

### 2.10 Immunohistochemistry

Immunohistochemical staining was performed according to standard protocols with minor modifications (Oh et al., 2024; Ha et al., 2025; Oh et al., 2025). Briefly, free-floating coronal brain sections were incubated in 3% hydrogen peroxide for 30 min to quench endogenous peroxidase activity. After washing in phosphate-buffered saline (PBS) three times for 10 min each, sections were blocked with a solution containing 5% normal goat serum, 2% bovine serum albumin, 2% fetal bovine serum, and 0.1% Triton X-100 in PBS for 1 h at room temperature.

Tissues were incubated overnight (≥18 h) at room temperature with the following primary antibodies: mouse anti-PECAM-1/CD31 (1:500; Santa Cruz Biotechnology, Dallas, TX, United States) and rabbit anti-Iba-1 (1:2000; Wako Pure Chemical Industries, Osaka, Japan). After washing, sections were sequentially incubated with biotinylated secondary antibodies—rabbit anti-mouse IgG or goat anti-rabbit IgG (1:250; Vector Laboratories, Burlingame, CA, United States)—and the avidin–biotin complex (ABC Kit; Vector Laboratories, Burlingame, CA, United States) for 1 h each at room temperature.

Immunoreactivity was visualized using 3,3′-diaminobenzidine (DAB) in Tris buffer (pH 7.4) for 3–5 min. Stained sections were mounted onto gelatin-coated slides, air-dried, dehydrated through graded ethanol, cleared in xylene, and cover-slipped. Immunostaining was examined under a light microscope.

### 2.11 Quantification of immunohisto chemical staining

Quantification of Iba-1 immunoreactivity was performed based on previously established methods (Oh et al., 2024; Ha et al., 2025; Oh et al., 2025). Briefly, immunopositive signals were captured using a DP70 imaging system (Olympus, Tokyo, Japan) at three predefined regions: the infarct border, the ipsilateral proximal region adjacent to the infarct, and the ipsilateral distal region farther from the infarct. Image analysis was conducted using NIH ImageJ software (http://rsbweb.nih.gov/ij/) to measure the optical density of Iba-1–positive areas. The relative density values were used as an index of microglial activation.

### 2.12 BV2 cell culture

BV2 cells, a murine microglial cell line, were obtained from the School of Dentistry, Seoul National University, and cultured according to previously established protocols (Choi et al., 2023; Jang et al., 2023; Jo et al., 2024). Briefly, cells were maintained in 100-mm culture dishes containing Dulbecco’s Modified Eagle’s Medium (DMEM; Gibco, Grand Island, NY, United States) supplemented with 10% fetal bovine serum (FBS; Gibco) and 1% penicillin-streptomycin (P/S; Gibco). Cultures were incubated at 37 °C in a humidified atmosphere of 5% CO_2_ and the culture medium was replaced every 3 days.

### 2.13 bEND.3 cell culture and CHD treatment

All procedures were performed in accordance with previously established methods, with slight modifications (Oh et al., 2024; Oh et al., 2025). Briefly, bEND.3 cells (ATCC® CRL-2299™), a mouse brain microvascular endothelial cell line, were cultured in Dulbecco’s Modified Eagle Medium (DMEM) supplemented with 10% fetal bovine serum (FBS) and 1% penicillin–streptomycin (Gibco, Thermo Fisher Scientific, Waltham, MA, United States) at 37 °C in a humidified incubator containing 5% CO_2_. After stabilization, cells were seeded in 6-well plates at a density of 3 × 10^5^ cells/well and allowed to attach for 24 h. To model BBB disruption, lipopolysaccharide (LPS; 1 μg/mL) was added to the medium for 4 h. CHD was administered at concentrations of 1, 10, or 100 μg/mL for 1 h prior to LPS exposure.

### 2.14 MTT assay

The cytotoxicity of CHD was assessed in BV2 microglial cells using the MTT [3-(4,5-dimethylthiazol-2-yl)-2,5-diphenyltetrazolium bromide] assay, based on previously reported methods (Jo et al., 2024). Briefly, BV2 cells were seeded into 96-well plates and treated with various concentrations of CHD (0.001–400 μg/mL). After 24 h of incubation, MTT solution (10 μL per 100 μL medium) was added to each well and incubated for 4 h at 37 °C. The supernatant was then removed, and 100 μL of DMSO was added to dissolve the formazan crystals. Absorbance was measured at 595 nm using a microplate spectrophotometer. The MTT assay was additionally performed in bEND.3 endothelial cells to determine the non-cytotoxic concentration range of CHD prior to Western blot analysis.

### 2.15 Western blotting

Protein expression levels in BV2 cells were assessed *via* Western blotting, following standard protocols with minor modifications (Choi et al., 2023; Jang et al., 2023; Jo et al., 2024). Briefly, after treatment with CHD (at selected concentrations) for 24 h, cells were lysed using RIPA buffer supplemented with protease and phosphatase inhibitors. Protein concentrations were quantified using a BCA assay, and equal amounts of protein were separated by SDS-PAGE and transferred onto PVDF membranes. Membranes were blocked with 5% skim milk in TBST and incubated overnight at 4 °C with primary antibodies against target proteins (e.g., NLRP3, Caspase-1, IL-1β, and β-actin). After washing, membranes were incubated with HRP-conjugated secondary antibodies for 1 h at room temperature. Signals were detected using enhanced chemiluminescence (ECL) and visualized with a ChemiDoc imaging system. Band intensities were quantified using ImageJ software and normalized to β-actin.

### 2.16 Statistical analysis

All data are presented as mean ± standard deviation (SD). Statistical analyses were performed using GraphPad Prism (version X; GraphPad Software Inc., San Diego, CA, United States). One-way ANOVA followed by Tukey’s *post hoc* test was used for comparisons among three or more independent groups. For comparisons between two groups, an unpaired two-tailed Student’s t-test was applied. For cerebral blood flow (rCBF) measurements obtained by laser Doppler flowmetry, two-way repeated-measures ANOVA followed by Bonferroni’s multiple-comparisons test was used to evaluate the effects of treatment and time. A *p*-value <0.05 was considered statistically significant. All experiments were performed at least in triplicate unless otherwise stated. The number of animals or replicates (n) for each experiment is specified in the figure legends. Outliers were not excluded unless predetermined exclusion criteria were met, such as subarachnoid hemorrhage or failure to induce infarction in pMCAO models.

## 3 Results

### 3.1 Quality control of CHD extract

For quality control, the representative marker metabolites were quantitatively determined according to established methods (Jung et al., 2018; Lee et al., 2023): geniposide from *Gardeniae Fructus,* sennoside A from *Rhei Rhizoma,* coptisine from *Coptidis Rhizoma,* berberine from *Phellodendri Cortex,* wogonin from *Scutellariae Radix.* To ensure reproducibility and batch-to-batch consistency, the CHD extract was analyzed by high-performance liquid chromatography (HPLC). Quantified contents (mg/g extract) were as follows: geniposide (21.2 min, 41.14 mg/g), sennoside A (25.7 min, 14.35 mg/g), coptisine (27.8 min, 1.39 mg/g), berberine (29.7 min, 8.93 mg/g), and wogonin (39.9 min, 10.20 mg/g) (Figure 1). This profiling confirmed the presence of representative alkaloids, flavonoids, and iridoid glycosides, thereby establishing a reproducible phytochemical fingerprint profile for batch-level quality control for use in the present study.

### 3.2 CHD reduces cortical infarct volume in a mouse model of ischemic stroke

**FIGURE 2:**
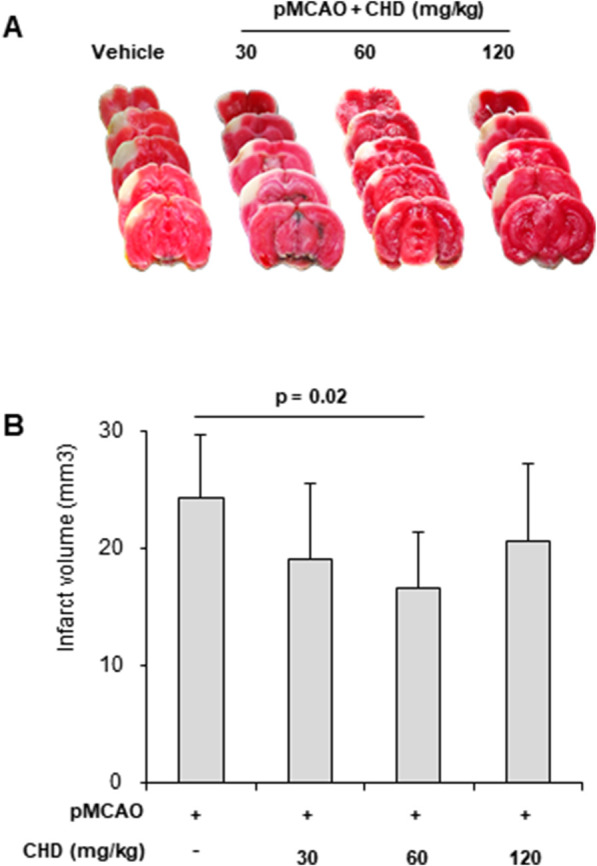
CHD reduces cortical infarct volume after permanent MCA occlusion (pMCAO). **(A)** Representative TTC-stained coronal brain sections obtained 24 h after pMCAO. Pale regions indicate infarcted cortex. Vehicle-treated mice showed extensive cortical infarction, whereas CHD administration (30, 60, or 120 mg/kg, p.o., 10 min prior to occlusion) reduced infarct size. **(B)** Quantitative analysis of cortical infarct volume at 24 h post-pMCAO in vehicle-treated mice (DW, *n* = 11) and CHD-treated groups (30 mg/kg, *n* = 11; 60 mg/kg, *n* = 11; 120 mg/kg, *n* = 10). CHD significantly reduced infarct volume at 60 mg/kg, with other doses showing a decreasing trend compared with vehicle. Data are presented as mean ± SD. Statistical analysis was performed using one-way ANOVA followed by Tukey’s *post hoc* test.

To evaluate the neuroprotective efficacy of CHD, infarct volume was measured 24 h after pMCAO using TTC staining (Figure 2). Vehicle-treated mice (*n* = 11) exhibited extensive cortical infarction with marked pale discoloration, whereas CHD administration at 30 mg/kg (*n* = 11), 60 mg/kg (*n* = 11), and 120 mg/kg (*n* = 10) reduced infarct size in a dose-dependent manner (Figure 2A). Quantitative analysis confirmed a significant reduction in cortical infarct volume in the 60 mg/kg group (vehicle: 24.2 ± 5.4 mm^3^; CHD 60 mg/kg: 16.6 ± 4.8 mm^3^; *p* < 0.05), while both 30 mg/kg and 120 mg/kg groups also showed a trend toward decreased infarct volume compared with vehicle (Figures 2A,B). These findings demonstrate that CHD confers potent neuroprotection against ischemic injury, likely through improved collateral perfusion, suppression of neuroinflammation, and stabilization of vascular integrity.

### 3.3 CHD enhances ACA perfusion following MCA occlusion

**FIGURE 3:**
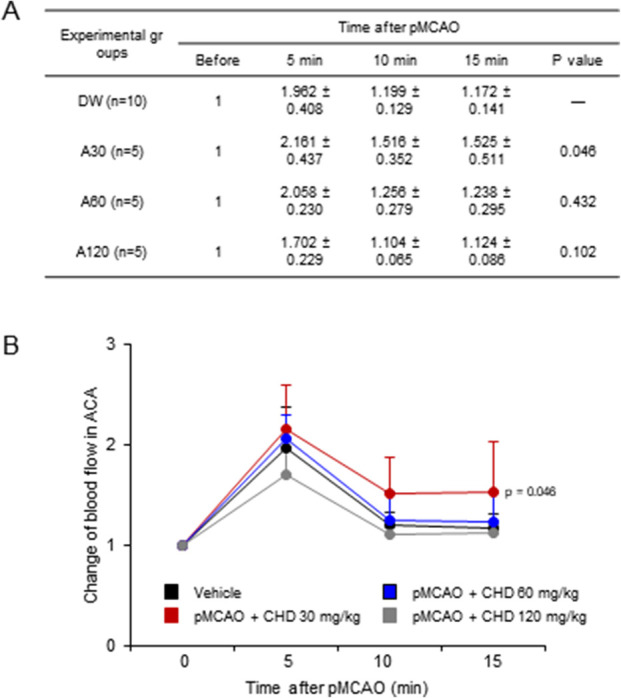
Effects of CHD on regional cerebral blood flow in the ACA territory following permanent MCA occlusion (pMCAO). **(A)** Summary table of regional cerebral blood flow (rCBF) in the ACA territory measured before and at 5, 10, and 15 min after pMCAO. Values are expressed as fold change relative to baseline (pre-occlusio*n* = 1). **(B)** Time-course analysis of rCBF changes in the ACA territory. CHD at 30 mg/kg increased rCBF compared with vehicle (*p* = 0.046), whereas no statistically significant differences were observed in the 60 mg/kg and 120 mg/kg groups. Data are presented as mean ± SD (*n* = 5–10 per group). Cerebral blood flow (rCBF) was analyzed using two-way repeated-measures ANOVA to assess the effects of treatment and time, followed by Bonferroni’s *post hoc* test for multiple comparisons. Exact *P* values are indicated in the figure.

Regional cerebral blood flow (rCBF) in the ACA territory was monitored after pMCAO (Figure 3). In vehicle-treated mice, rCBF transiently increased at 5 min (1.96 ± 0.41-fold) but subsequently declined (1.20 ± 0.13 at 10 min; 1.17 ± 0.14 at 15 min). In contrast, CHD at 30 mg/kg significantly enhanced perfusion, reaching 2.16 ± 0.44 at 5 min and remaining elevated at 10 min (1.52 ± 0.35, *p* = 0.046 vs. vehicle) and 15 min (1.53 ± 0.51, *p* = 0.046 vs. vehicle) (Figures 3A–C). Neither the intermediate dose (60 mg/kg) nor the higher dose (120 mg/kg) produced significant changes (Figures 3A–C). These findings indicate that CHD promotes redistribution of cerebral perfusion *via* leptomeningeal collaterals during early ischemia, thereby contributing to the observed reduction in infarct volume. Representative Doppler traces are provided in Supplementary Figure S1.

### 3.4 CHD promotes VEGF-mediated angiogenesis in the ischemic cortex and endothelial cells

**FIGURE 4:**
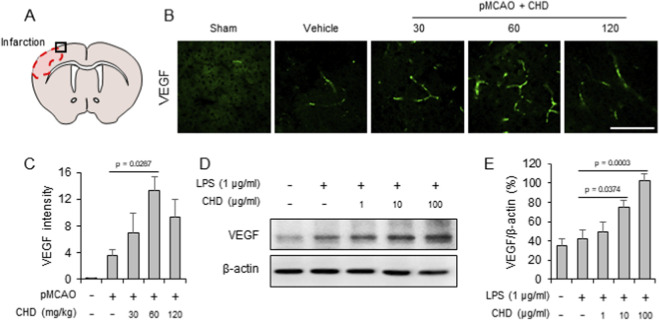
CHD promotes VEGF-mediated angiogenesis in ischemic cortex and endothelial cells. **(A)** Schematic illustration of coronal brain section indicating the peri-infarct region analyzed. **(B)** Representative immunofluorescence staining of VEGF in the peri-infarct cortex 24 h after pMCAO. VEGF expression was increased by pMCAO and further enhanced by CHD, particularly at 60 mg/kg. Scale bar = 50 μm. **(C)** Quantification of VEGF fluorescence intensity in (B), showing significant dose-dependent enhancement by CHD. **(D)** Western blot analysis of VEGF protein levels in LPS-stimulated bEND.3 endothelial cells treated with CHD (1–100 μg/mL, 1 h pretreatment). **(E)** Densitometric quantification of (D), normalized to β-actin, confirming dose-dependent upregulation of VEGF. Data are presented as mean ± SD. Statistical analysis was performed using one-way ANOVA followed by Tukey’s *post hoc* test.

Prior to VEGF analysis, cell viability was assessed using an MTT assay to determine non-cytotoxic concentrations of CHD in bEND.3 cells. CHD concentrations up to 100 μg/mL did not significantly reduce cell viability (Supplementary Figure S2), and these concentrations were therefore selected for subsequent experiments. To investigate whether the CHD-induced increase in cerebral perfusion was associated with angiogenic remodeling, VEGF expression was examined in the ischemic cortex (Figure 4). Immunofluorescence analysis revealed increased VEGF immunoreactivity in the peri-infarct region of vehicle-treated mice, which was further augmented by CHD administration, with the most pronounced effect observed at 60 mg/kg (Figures 4A–C). *In vitro*, LPS-stimulated bEND.3 endothelial cells exhibited modest upregulation of VEGF, whereas co-treatment with CHD (1–100 μg/mL) markedly increased VEGF protein expression in a concentration-responsive manner, as shown by Western blot and densitometric analysis (Figures 4D,E). Together, these findings suggest that CHD facilitates endogenous angiogenesis, potentially enhancing the development and/or dilation of collateral vessels in response to ischemic stress.

### 3.5 CHD suppresses neuroinflammation by inhibiting microglial activation and pro-inflammatory mediators

**FIGURE 5:**
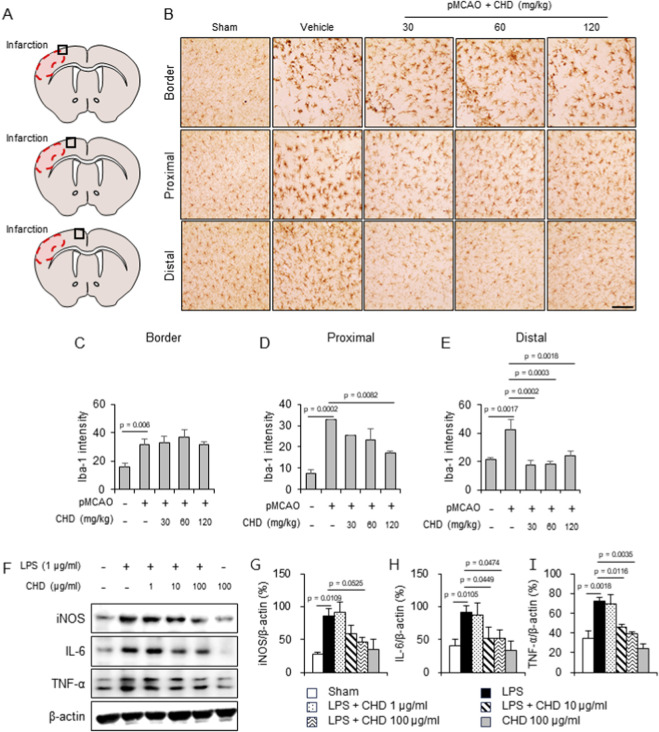
CHD suppresses microglial activation and pro-inflammatory mediator expression. **(A–E)** Immunohistochemistry for Iba-1 in peri-infarct cortex 24 h after pMCAO. Vehicle-treated mice exhibited increased Iba-1–positive microglia in proximal and distal regions, whereas CHD administration (30, 60, and 120 mg/kg, p.o.) significantly attenuated microglial activation. Scale bar = 100 μm. **(F–I)** Western blot analyses of iNOS, IL-6, and TNF-α in BV2 microglial cells pretreated with CHD (10–100 μg/mL, 1 h) followed by LPS stimulation (1 μg/mL, 4 h) **(F)**. Densitometric quantification normalized to β-actin, showing concentration-responsive suppression of inflammatory mediator expression **(G–I)**. Data are presented as mean ± SD. Statistical significance was determined by one-way ANOVA followed by Tukey’s *post hoc* test. Exact p values are indicated in the graphs.

To assess the anti-inflammatory effects of CHD after ischemic injury, immunohistochemical staining for Iba-1, a marker of microglial activation, was performed in peri-infarct cortical regions (Figure 5). Vehicle-treated mice exhibited a marked increase in Iba-1–positive microglia, particularly in proximal and distal areas relative to the infarct border. CHD administration significantly attenuated Iba-1 immunoreactivity in these regions, whereas no significant effect was observed at the infarct border zone (Figures 5A–E). To exclude potential cytotoxic effects, BV2 cell viability was evaluated by MTT assay. CHD concentrations up to 100 μg/mL did not significantly affect cell viability, whereas higher concentrations reduced viability (Supplementary Figure S3). In line with the *in vivo* findings, LPS-stimulated BV2 microglial cells showed robust upregulation of inflammatory proteins, including iNOS, IL-6, and TNF-α. Pretreatment with CHD (10–100 μg/mL) suppressed the expression of these mediators in a concentration-responsive manner within the tested range (Figures 5F–I). Collectively, these findings indicate that CHD mitigates ischemia-induced neuroinflammation by inhibiting microglial activation and restraining the production of pro-inflammatory cytokines and enzymes.

### 3.6 CHD preserves endothelial barrier integrity by modulating junctional proteins and STAT3 signaling

**FIGURE 6:**
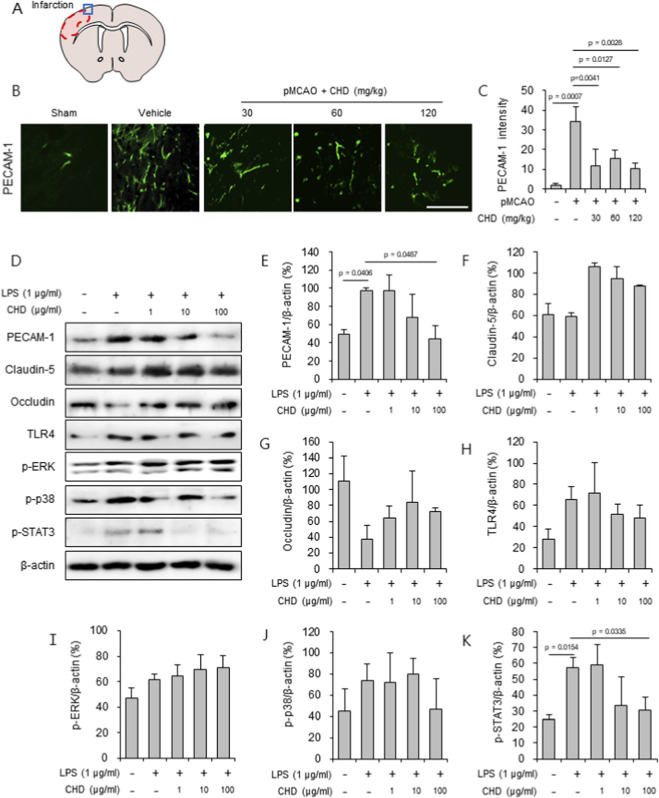
CHD preserves endothelial barrier integrity by modulating junctional proteins and TLR4/STAT3 signaling. **(A)** Schematic showing infarct region used for analysis. **(B,C)** PECAM-1 immunofluorescence in peri-infarct cortex 24 h after pMCAO. Vehicle-treated mice exhibited strong PECAM-1 expression, which was significantly reduced by CHD (30, 60, 120 mg/kg, p.o.). Scale bar = 50 μm. **(D–K)** Western blot analyses of PECAM-1, claudin-5, occludin, TLR4, p-ERK, p-p38, and p-STAT3 in LPS-stimulated bEND.3 endothelial cells with or without CHD pretreatment (1–100 μg/mL) (D). Densitometric quantification normalized to β-actin. CHD dose-dependently suppressed PECAM-1 expression, restored claudin-5, and selectively inhibited p-STAT3, whereas TLR4, p-ERK, and p-p38 remained unchanged **(E–K)**. Representative Western blots are shown. Densitometric analysis was normalized to β-actin. Data are presented as mean ± SD. Statistical significance was determined by one-way ANOVA followed by Tukey’s *post hoc* test. Exact *p* values are indicated in the graphs.

To investigate whether CHD protects against ischemia-induced endothelial dysfunction, PECAM-1 immunofluorescence was examined in the peri-infarct cortex (Figure 6). Vehicle-treated mice exhibited markedly elevated PECAM-1 expression, consistent with endothelial activation and blood–brain barrier (BBB) disruption. CHD significantly reduced PECAM-1 levels across all tested doses (30, 60, and 120 mg/kg) (Figures 6B,C). To further evaluate these effects, LPS-stimulated bEND.3 endothelial cells were analyzed by Western blotting (Figures 6D–K). CHD pretreatment (1–100 μg/mL) dose-dependently reversed LPS-induced upregulation of PECAM-1 and restored the expression of the tight junction proteins claudin-5 (Figures 6D–G). Mechanistically, CHD suppressed phosphorylated STAT3 (p-STAT3), whereas TLR4, p-ERK and p-p38 levels were unaffected (Figures 6H–K), suggesting selective inhibition of the STAT3 signaling pathway. Collectively, these results indicate that CHD preserves endothelial barrier integrity by attenuating inflammatory signaling—particularly through STAT3 suppression—and stabilizing junctional architecture within the neurovascular unit.

## 4 Discussion

This study provides the first experimental evidence that CHD, a traditional multi-botanical extract, enhances collateral circulation and protects the neurovascular unit in a mouse model of acute ischemic stroke. CHD significantly reduced cortical infarct volume, increased ACA perfusion, promoted angiogenesis, suppressed neuroinflammation, and preserved blood–brain barrier (BBB) integrity. These findings indicate that CHD may serve as a promising pharmacological strategy for targeting collateral circulation, a therapeutic gap in current stroke treatment paradigms.

### 4.1 Collateral perfusion and VEGF-mediated vascular adaptation

Collateral circulation is increasingly recognized as a critical determinant of infarct size, functional recovery, and therapeutic responsiveness in ischemic stroke (Maguida and Shuaib, 2023; Liu et al., 2025; Marilena et al., 2025). In this study, CHD rapidly increased ACA blood flow following pMCAO, as confirmed by laser Doppler flowmetry. This early perfusion enhancement likely reflects improved leptomeningeal anastomosis flow, which sustains penumbral viability and delays infarct progression (Vasquez et al., 2021; Marilena et al., 2025). Mechanistically, CHD upregulated VEGF expression in ischemic brain tissue and LPS-stimulated endothelial cells (Figure 4). VEGF is a central mediator of angiogenesis and arteriogenesis—key processes in collateral remodeling (Clayton et al., 2008; Eelen et al., 2020). Together, these findings suggest that CHD supports both acute vascular responses and longer-term vascular adaptation.

### 4.2 Multi-pathway signaling integration and dose-dependent neurovascular outcomes

Although ERK and p38 are recognized downstream mediators of VEGF signaling, their activation is highly time-dependent and typically transient, often peaking within minutes to hours after stimulation (Simons et al., 2016). Because protein expression in the present study was assessed at a later time point under ischemic conditions, early phosphorylation events may not have been detected. Therefore, the absence of sustained p-ERK and p-p38 elevation does not necessarily negate upstream VEGF pathway activation. Moreover, VEGF-driven angiogenesis is regulated through multiple parallel cascades—including PI3K/Akt and nitric oxide–related signaling—highlighting coordinated multi-pathway integration rather than reliance on a single MAPK axis (Carmeliet and Jain, 2011; Potente and Makinen, 2017). The dose-dependent dissociation observed in this study likely reflects temporally distinct mechanisms governing early collateral perfusion and ultimate infarct limitation. CHD at 30 mg/kg significantly enhanced early ACA perfusion following MCA occlusion; however, this transient hemodynamic improvement alone was insufficient to reduce infarct volume at 24 h. In contrast, the 60 mg/kg dose significantly reduced infarct size without further augmenting early ACA flow, indicating that sustained neurovascular protection extends beyond immediate perfusion effects. At this dose, CHD enhanced VEGF expression, suppressed microglial activation, and preserved endothelial junctional integrity, collectively supporting durable tissue protection. Thus, early collateral perfusion and final infarct reduction represent related yet mechanistically distinct outcomes, reflecting multi-level neurovascular regulation rather than a singular hemodynamic mechanism. Importantly, no evidence of dose-related toxicity was observed at the highest tested dose (120 mg/kg). The highest dose did not worsen infarct size, inflammatory markers, or endothelial injury compared with the vehicle group, and no mortality or abnormal behavioral changes were detected during the experimental period. These findings indicate that the non-linear dose–response pattern reflects differential engagement or saturation of protective mechanisms rather than toxicity. Consistent with this interpretation, prior clinical evaluation involving 656 patients reported a low incidence of adverse events (2.0%) and no evidence of hepatotoxicity or nephrotoxicity (Cho et al., 2003), further supporting the safety profile of CHD within clinically relevant dosage ranges.

### 4.3 Neurovascular protection via anti-inflammatory and endothelial-stabilizing effects

Post-ischemic neuroinflammation and endothelial barrier dysfunction exacerbate secondary brain injury (Candelario-Jalil et al., 2022; Duan et al., 2024). CHD significantly reduced Iba-1–positive microglial activation in peri-infarct regions and suppressed the expression of iNOS, IL-6, and TNF-α. These anti-inflammatory effects are consistent with prior reports showing that constituents found in CHD (e.g., berberine, baicalin, and wogonin) can modulate inflammatory signaling and oxidative stress responses (Shieh et al., 2000; Qiu et al., 2025). Moreover, CHD preserved BBB integrity by reducing PECAM-1 expression and restoring tight junction proteins such as claudin-5. PECAM-1 is not only a marker but also a mediator of vascular inflammation and permeability (Privratsky et al., 2010). Stabilization of the endothelial barrier likely limited leukocyte infiltration and vasogenic edema, both of which contribute to infarct expansion (Chen et al., 2021; Stanzione et al., 2022).

### 4.4 CHD suppresses STAT3 signaling to preserve endothelial integrity

Endothelial dysfunction and inflammation are key contributors to BBB disruption after ischemic stroke (Kadry et al., 2020; Zapata-Acevedo et al., 2024). In the present study, CHD preserved endothelial integrity by selectively downregulating p-STAT3, whereas TLR4, p-ERK, and p-p38 remained largely unaffected. STAT3 is an established regulator of endothelial activation and barrier breakdown in neuroinflammatory conditions (Narasimhan et al., 2009; Gesuete et al., 2014; Wang et al., 2015; Mowry et al., 2021; Huang et al., 2026; Wu et al., 2025), suggesting that selective STAT3 suppression represents a principal mechanism underlying CHD-mediated barrier stabilization. Although certain CHD constituents have been reported to influence TLR4/MAPK signaling in macrophage-dominant inflammatory models, endothelial signaling responses are highly context- and time-dependent. MAPK phosphorylation is typically rapid and transient following receptor stimulation, and early dynamic changes may not persist at later time points (Carmeliet and Jain, 2011; Simons et al., 2016). Moreover, endothelial regulation involves coordinated multi-pathway signaling—including PI3K/Akt, eNOS, and STAT pathways—rather than reliance on a single MAPK cascade (De Bock et al., 2013; Carmeliet and Jain, 2011; Potente and Makinen, 2017). Within this framework, the observed selectivity toward STAT3 likely reflects preferential engagement of barrier-regulatory mechanisms rather than broad upstream inhibition of the TLR4/MAPK axis.

### 4.5 Therapeutic implications and translational potential

Currently, therapeutic approaches to enhance collateral circulation are limited to mechanical or investigational methods, with no approved drugs directly targeting this vascular system (Marilena et al., 2025). The present study suggests that CHD—through its multifaceted effects on cerebral perfusion, angiogenesis, inflammation, and BBB stabilization—can address this unmet need. Moreover, oral administration, rapid efficacy, and reported clinical tolerability of CHD (Jung et al., 2018; Lee et al., 2023) enhance its translational potential for ischemic stroke treatment.

### 4.6 Limitations and future directions

This study primarily examined acute-phase neuroprotection; its influence on long-term neurological and functional outcomes requires extended follow-up studies. Although STAT3 inhibition emerged as a central regulatory mechanism, additional pathways—such as eNOS/PI3K/Akt, alternative MAPK cascades, and VEGF-associated downstream effectors—may also contribute to CHD’s effects. Comprehensive multi-pathway and time-resolved analyses will be essential to delineate the broader molecular network. Furthermore, as CHD is a multi-component botanical formulation, the independent efficacy, synergistic interactions, and pharmacokinetic/pharmacodynamic characteristics of its bioactive constituents remain to be defined. Future studies isolating and mechanistically evaluating key compounds will be critical for robust quality control, formulation optimization, and clinical translation.

## 5 Conclusion

CHD exerts multi-level neurovascular protection in ischemic stroke by enhancing collateral perfusion and selectively suppressing STAT3-dependent endothelial inflammatory signaling. These findings support the therapeutic potential of CHD as a pharmacological modulator of collateral circulation and neurovascular stability and warrant further translational and clinical investigation.

### 5.1 Limitations of the study

No positive control was included in this analysis. Based on the authors’ assessment, there is currently no established pharmacological agent specifically targeting collateral perfusion in ischemic stroke. Therefore, this study was designed to evaluate the mechanistic effects of CHD relative to vehicle treatment rather than to compare it with an established collateral-targeting agent.

## Data Availability

The original contributions presented in the study are included in the article/Supplementary Material, further inquiries can be directed to the corresponding authors.

## References

[B1] Candelario-Jalil E. Dijkhuizen R. M. Magnus T. (2022). Neuroinflammation, stroke, blood-brain barrier dysfunction, and imaging modalities. Stroke 53, 1473–1486. 10.1161/STROKEAHA.122.036946 35387495 PMC9038693

[B2] Carmeliet P. Jain R. K. (2011). Molecular mechanisms and clinical applications of angiogenesis. Nature 473, 298–307. 10.1038/nature10144 21593862 PMC4049445

[B3] Chen S. Shao L. Ma L. (2021). Cerebral Edema formation after stroke: emphasis on blood-brain barrier and the lymphatic drainage system of the brain. Front. Cell Neurosci. 15, 716825. 10.3389/fncel.2021.716825 34483842 PMC8415457

[B4] Cho K. H. Jung W. S. Park S. W. Moon S. K. Kim Y. S. Hae H. S. (2003). Clinical assessment on the safety of Chunghyul-dan (Qingwie-dan). J. Korean Orient Med. 24, 45–50.

[B5] Choi W. W. Lee K. Lee B. J. Park S. U. Park J. M. Ko C. N. (2017). The effects of Chunghyul-Dan, an agent of Korean medicine, on a mouse model of traumatic brain injury. Evid. Based Complement. Altern. Med. 2017, 7326107. 10.1155/2017/7326107 28684970 PMC5480248

[B6] Choi J. H. Kwon T. W. Jo H. S. Ha Y. Cho I. H. (2023). Gintonin, a *Panax ginsen*g-derived LPA receptor ligand, attenuates kainic acid-induced seizures and neuronal cell death in the hippocampus *via* anti-inflammatory and anti-oxidant activities. J. Ginseng Res. 47, 390–399. 10.1016/j.jgr.2022.11.001 37252272 PMC10214296

[B7] Clayton J. A. Chalothorn D. Faber J. E. (2008). Vascular endothelial growth factor-A specifies formation of native collaterals and regulates collateral growth in ischemia. Circ. Res. 103, 1027–1036. 10.1161/CIRCRESAHA.108.181115 18802023 PMC2729271

[B8] Collaborators G. B. D. S. R. F. (2024). Global, regional, and national burden of stroke and its risk factors, 1990–2021: a systematic analysis for the global Burden of Disease Study 2021. Lancet Neurol. 23, 973–1003. 10.1016/S1474-4422(24)00369-7 39304265 PMC12254192

[B9] De Bock K. Georgiadou M. Carmeliet P. (2013). Role of endothelial cell metabolism in vessel sprouting. Cell Metab. 18, 634–647. 10.1016/j.cmet.2013.08.001 23973331

[B10] Duan M. Xu Y. Li Y. Feng H. Chen Y. (2024). Targeting brain-peripheral immune responses for secondary brain injury after ischemic and hemorrhagic stroke. J. Neuroinflammation 21, 102. 10.1186/s12974-024-03101-y 38637850 PMC11025216

[B11] Eelen G. Treps L. Li X. Carmeliet P. (2020). Basic and therapeutic aspects of angiogenesis updated. Circ. Res. 127, 310–329. 10.1161/CIRCRESAHA.120.316851 32833569

[B12] Fan J. L. Brassard P. Rickards C. A. Nogueira R. C. Nasr N. Mcbryde F. D. (2022). Integrative cerebral blood flow regulation in ischemic stroke. J. Cereb. Blood Flow. Metab. 42, 387–403. 10.1177/0271678X211032029 34259070 PMC8985438

[B13] Fan S. Yang L. Ji X. (2025). Reperfusion therapy for acute ischemic stroke: where we are and where to go. J. Transl. Int. Med. 13, 1–3. 10.1515/jtim-2025-0001 40115035 PMC11921817

[B14] Gesuete R. Kohama S. G. Stenzel-Poore M. P. (2014). Toll-like receptors and ischemic brain injury. J. Neuropathol. Exp. Neurol. 73, 378–386. 10.1097/NEN.0000000000000068 24709682 PMC4115675

[B15] Groan M. Ospel J. Ajmi S. Sandset E. C. Kurz M. W. Skjelland M. (2021). Time-based decision making for reperfusion in acute ischemic stroke. Front. Neurol. 12, 728012. 10.3389/fneur.2021.728012 34790159 PMC8591257

[B16] Ha Y. Jo H. S. Kwon T. W. Jeon S. H. Moon S. K. Jung J. H. (2025). Korean black ginseng extract alleviates Alzheimer’s disease-related cognitive impairment by activating the Nrf2/HO-1 pathway and suppressing the p38 MAPK/NF-kappaB/STAT3 pathways and NLRP3 inflammasome *via* TLR2 and TLR4 modulation. J. Ginseng Res. 49, 294–305. 10.1016/j.jgr.2025.02.002 40453349 PMC12125584

[B18] Huang C. Mo X. Liu Y. Hu D. Li C. Zhao Y. (2026). NFKBIZ mediates neuroprotection and maintains blood-brain barrier integrity in cerebral ischemia/reperfusion *via* STAT3-regulated Nrf2/ARE signaling. Exp. Neurol. 398, 115638. 10.1016/j.expneurol.2026.115638 41513081

[B19] Jang M. Choi J. H. Jang D. S. Cho I. H. (2023). Micrandilactone C, a nortriterpenoid isolated from roots of *Schisandra chinensis*, ameliorates Huntington’s disease by inhibiting microglial STAT3 pathways. Cells 12, 786. 10.3390/cells12050786 36899922 PMC10000367

[B20] Jin Z. Lan Y. Li J. Wang P. Xiong X. (2024). The role of Chinese herbal medicine in the regulation of oxidative stress in treating hypertension: from therapeutics to mechanisms. Chin. Med. 19, 150. 10.1186/s13020-024-01022-9 39468572 PMC11520704

[B21] Jo H. S. Lee Y. W. Son S. R. Jang D. S. Woo Kwon T. Ha Y. (2024). A supercritical oil extract of *Schisandra chinensis* seeds ameliorates Huntington’s disease-like symptoms and neuropathology: the potential role of anti-oxidant and anti-inflammatory effects. Front. Pharmacol. 15, 1471024. 10.3389/fphar.2024.1471024 39764462 PMC11700980

[B22] Jung W. S. Min I. K. Jin C. Park J. Y. Kim H. G. Kwak Y. (2018). Inhibitory effect of Chunghyul-dan on stroke recurrence in small vessel disease patients: a 5-year observational Study. J. Evid. Based Integr. Med. 23, 2515690X18789374. 10.1177/2515690X18789374 30045628 PMC6073819

[B23] Kadry H. Noorani B. Cucullo L. (2020). A blood-brain barrier overview on structure, function, impairment, and biomarkers of integrity. Fluids Barriers CNS 17, 69. 10.1186/s12987-020-00230-3 33208141 PMC7672931

[B24] Kim Y. S. Jung E. A. Shin J. E. Chang J. C. Yang H. K. Kim N. J. (2002). Daio-Orengedokuto inhibits HMG-CoA reductase and pancreatic lipase. Biol. Pharm. Bull. 25, 1442–1445. 10.1248/bpb.25.1442 12419956

[B25] Kim H. G. Ju M. S. Kim D. H. Hong J. Cho S. H. Cho K. H. (2010). Protective effects of Chunghyuldan against ROS-mediated neuronal cell death in models of Parkinson’s disease. Basic Clin. Pharmacol. Toxicol. 107, 958–964. 10.1111/j.1742-7843.2010.00612.x 20629656

[B26] Landis S. C. Amara S. G. Asadullah K. Austin C. P. Blumenstein R. Bradley E. W. (2012). A call for transparent reporting to optimize the predictive value of preclinical research. Nature 490, 187–191. 10.1038/nature11556 23060188 PMC3511845

[B27] Lee B. Kwon C. Y. (2020). Effectiveness and safety of Hwangryunhaedok-Tang (Huang-Lian-Jie-Du-Tang, Oren-Gedoku-to) for dyslipidemia: a protocol for a PRISMA-compliant systematic review and meta-analysis. Med. Baltim. 99, e23367. 10.1097/MD.0000000000023367 33371067 PMC7748370

[B28] Lee H. G. Kwon S. Cho S. Y. Park S. U. Jung W. S. Moon S. K. (2023). Effect of an herbal medicine, Chunghyul-dan, on prevention of recurrence in patients with ischemic stroke: a retrospective cohort study. Med. Baltim. 102, e35840. 10.1097/MD.0000000000035840 37960767 PMC10637502

[B29] Li R. (2016). Natural product-based drug discovery. Med. Res. Rev. 36, 3. 10.1002/med.21380 26662060

[B30] Liu H. Yao N. Hu H. Xiao Z. Tang Y. Tang S. (2025). Collateral circulation significantly improves prognosis in cerebral infarction: a meta-analysis. Am. J. Transl. Res. 17, 4546–4561. 10.62347/XCXK6861 40672639 PMC12261181

[B31] Maguida G. Shuaib A. (2023). Collateral circulation in ischemic stroke: an updated review. J. Stroke 25, 179–198. 10.5853/jos.2022.02936 36907186 PMC10250877

[B32] Majid A. He Y. Y. Gidday J. M. Kaplan S. S. Gonzales E. R. Park T. S. (2000). Differences in vulnerability to permanent focal cerebral ischemia among 3 common mouse strains. Stroke 31, 2707–2714. 10.1161/01.str.31.11.2707 11062298

[B33] Marilena M. Romana P. F. Guido A. Gianluca R. Sebastiano F. Enrico P. (2025). From “time is brain” to “time is collaterals”: updates on the role of cerebral collateral circulation in stroke. J. Thromb. Thrombolysis. 58 (7), 821–832. 10.1007/s11239-025-03135-w 40544391

[B34] Mowry F. E. Peaden S. C. Stern J. E. Biancardi V. C. (2021). TLR4 and AT1R mediate blood-brain barrier disruption, neuroinflammation, and autonomic dysfunction in spontaneously hypertensive rats. Pharmacol. Res. 174, 105877. 10.1016/j.phrs.2021.105877 34610452 PMC8648989

[B35] Narasimhan P. Liu J. Song Y. S. Massengale J. L. Chan P. H. (2009). VEGF Stimulates the ERK 1/2 signaling pathway and apoptosis in cerebral endothelial cells after ischemic conditions. Stroke 40, 1467–1473. 10.1161/STROKEAHA.108.534644 19228841 PMC2663599

[B36] Oh J. Kwon T. W. Choi J. H. Kim Y. Moon S. K. Nah S. Y. (2024). Ginsenoside-Re inhibits experimental autoimmune encephalomyelitis as a mouse model of multiple sclerosis by downregulating TLR4/MyD88/NF-kappaB signaling pathways. Phytomedicine 122, 155065. 10.1016/j.phymed.2023.155065 37856989

[B37] Oh J. Ha Y. Kwon T. W. Jo H. S. Moon S. K. Lee Y. (2025). Non-saponin from *Panax ginseng* maintains blood-brain barrier integrity by inhibiting NF-kappaB and p38 MAP kinase signaling pathways to prevent the progression of experimental autoimmune encephalomyelitis. J. Ginseng Res. 49, 53–63. 10.1016/j.jgr.2024.09.005 39872290 PMC11764484

[B38] Potente M. Makinen T. (2017). Vascular heterogeneity and specialization in development and disease. Nat. Rev. Mol. Cell Biol. 18, 477–494. 10.1038/nrm.2017.36 28537573

[B39] Privratsky J. R. Newman D. K. Newman P. J. (2010). PECAM-1: conflicts of interest in inflammation. Life Sci. 87, 69–82. 10.1016/j.lfs.2010.06.001 20541560 PMC2917326

[B40] Qi Y. Zhang Q. Zhu H. (2019). Huang-Lian Jie-Du decoction: a review on phytochemical, pharmacological and pharmacokinetic investigations. Chin. Med. 14, 57. 10.1186/s13020-019-0277-2 31867052 PMC6918586

[B41] Qiu X. Huang R. Xie J. Luo S. Cheng X. Cui J. (2025). Recent advances in the therapeutic effects and molecular mechanisms of baicalin. Biol. (Basel) 14, 637. 10.3390/biology14060637 40563888 PMC12190052

[B42] Sarraj A. Yoshimura S. Thomalla G. Huo X. Arquizan C. Yoo A. J. (2025). Mechanical thrombectomy for large ischemic stroke: a critical appraisal of evidence from 6 randomized controlled trials. Stroke 56, 1917–1927. 10.1161/STROKEAHA.125.050402 40391430

[B43] Shieh D. E. Liu L. T. Lin C. C. (2000). Antioxidant and free radical scavenging effects of baicalein, baicalin and wogonin. Anticancer Res. 20, 2861–2865. 11062694

[B44] Simons M. Gordon E. Claesson-Welsh L. (2016). Mechanisms and regulation of endothelial VEGF receptor signalling. Nat. Rev. Mol. Cell Biol. 17, 611–625. 10.1038/nrm.2016.87 27461391

[B45] Stanzione R. Forte M. Cotugno M. Bianchi F. Marchitti S. Rubattu S. (2022). Role of DAMPs and of leukocytes infiltration in ischemic stroke: insights from animal models and translation to the human disease. Cell Mol. Neurobiol. 42, 545–556. 10.1007/s10571-020-00966-4 32996044 PMC11441194

[B17] Vasquez H. E. Murlimanju B. V. Shrivastava A. Yeider A. D. Andrei F. J. Ezequiel G. (2021). Intracranial collateral circulation and its role in neurovascular pathology. Egypt. J. Neurosurg. 36 (2) 10.1186/s41984-020-00095-6

[B46] Wang L. F. Li X. Gao Y. B. Wang S. M. Zhao L. Dong J. (2015). Activation of VEGF/Flk-1-ERK pathway induced blood-brain barrier injury after microwave exposure. Mol. Neurobiol. 52, 478–491. 10.1007/s12035-014-8848-9 25195697

[B47] Wu J. W. Wang B. X. Sha X. W. Wang P. Du S. Q. Lu X. J. (2025). Mechanistic insights and therapeutic prospects of targeting the signal transducer and activator of transcription 3 signaling pathway in cerebrovascular diseases. Biochem. Pharmacol. 242, 117375. 10.1016/j.bcp.2025.117375 41057127

[B48] Xiong X. Yang X. Liu Y. Zhang Y. Wang P. Wang J. (2013). Chinese herbal formulas for treating hypertension in traditional Chinese medicine: perspective of modern science. Hypertens. Res. 36, 570–579. 10.1038/hr.2013.18 23552514 PMC3703711

[B49] Xuan Zheng M. H. Bähr M. Tatenhorst L. Doeppner T. R. (2021). *Cerebral ischemia; chapter 11Treating cerebral ischemia: novel therapeutic strategies from experimental stroke research* Brisbane (AU). StatPearls. Available online at: 10.36255/exonpublications.cerebralischemia.2021.therapy. 34905315

[B50] Zapata-Acevedo J. F. Mantilla-Galindo A. Vargas-Sanchez K. Gonzalez-Reyes R. E. (2024). Blood-brain barrier biomarkers. Adv. Clin. Chem. 121, 1–88. 10.1016/bs.acc.2024.04.004 38797540

[B51] Zhu Y. Ouyang Z. Du H. Wang M. Wang J. Sun H. (2022). New opportunities and challenges of natural products research: when target identification meets single-cell multiomics. Acta Pharm. Sin. B 12, 4011–4039. 10.1016/j.apsb.2022.08.022 36386472 PMC9643300

