# Supplementary figures and images for: Chunghyul-dan, a multi-botanical ethanol extract, improves collateral perfusion and neurovascular stability in permanent focal cerebral ischemia
Source: Front Pharmacol. 2026 Apr 20;17:1736412. doi: 10.3389/fphar.2026.1736412 (PMC13136867; doi:10.3389/fphar.2026.1736412)

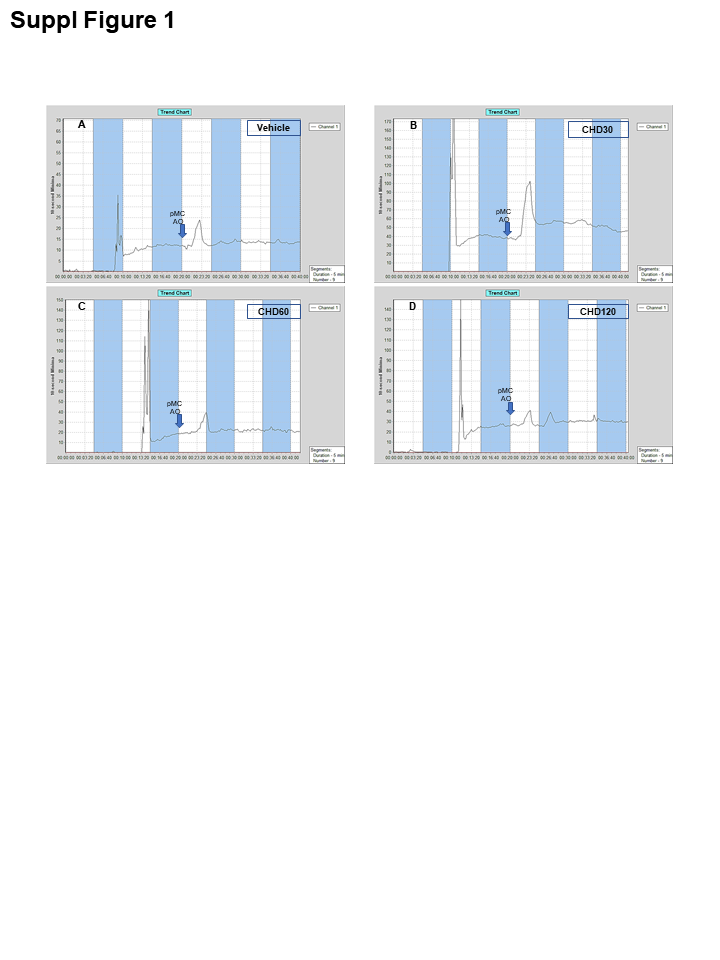

Supplement: Supplementary file 1 [file Image1.tif]
